# Radiological landscape of natural resources and mining: Unveiling the environmental impact of naturally occurring radioactive materials in Ghana's mining areas

**DOI:** 10.1016/j.heliyon.2024.e24959

**Published:** 2024-01-22

**Authors:** Augustine Faanu, Lordford Tettey-Larbi, Esther Osei Akuo-ko, Prince Kwabena Gyekye, David Okoh Kpeglo, Henry Lawluvi, Charles Kansaana, Serwaa Adjei-Kyereme, Alexander Opoku Efa, Edit Tóth-Bodrogi, Tibor Kovács, Amin Shahrokhi

**Affiliations:** a Radiological and Non-ionizing Installations Directorate, Nuclear Regulatory Authority, Ghana; b Radiation Protection Institute, Ghana Atomic Energy Commission, Ghana; c Research Centre for Biochemical, Environmental and Chemical Engineering, University of Pannonia, 8200, Veszprém, Hungary

**Keywords:** Mining, Gamma spectrometry, Statistical analysis, Environmental radioactivity, Radiation, Water, Soil, Public health

## Abstract

This study provides a general observation of the status of naturally occurring radioactive materials (NORMs) distribution in mining and industrial areas of Ghana in order to establish regional and national data on NORMs. The study includes data on radioactivity concentrations of U-238, Th-232, and K-40 in soils and for water concentrations of Ra-226, Th-228, and K-40 from various mining, oil, and gas communities, as well as water sources used for crop farming and farmlands. The average activity concentrations of U-238, Th-232, and K-40 in the soil samples were found to be 59 ± 16 Bq/kg, 48 ± 15 Bq/kg, and 286 ± 57 Bq/kg, respectively. The average concentration of Ra-226, Th-228, and K-40 in the water samples were found to be 1.62 ± 0.33 Bq/L, 2.08 ± 0.53 Bq/L, and 22.36 ± 3.44 Bq/L, respectively. The estimated average annual effective doses from external and internal exposure pathways in soil and water samples were 0.09 mSv/y and 0.54 mSv/y, respectively. The total annual effective dose resulting from both exposure pathways was calculated to be 0.63 mSv/y, which is below the 1 mSv/y dose limit recommended by the International Commission on Radiological Protection (ICRP) for controlling public radiation exposure. Based on the radiological hazard indices, the majority of the soil samples were found to be suitable as building materials as their respective indices were below the limits except for two sample locations and the sludge and scale samples. The average Excess Lifetime Cancer Risk (ELCR) value of the water samples was 1.6 times greater than the recommended value of 1.16 × 10^−3^, presenting a relatively higher risk to the public of developing cancer. No significant regional differences in the levels of radioactive elements. The regression models demonstrate strong interrelationships between the studied elements, with high R-squared values suggesting a predictable nature of one element’s concentration based on others.

## 1 Introduction

Natural radioactivity is the primary source of population dose [[1], [2], [3]]. The main contributors to external gamma radiation exposures are U-238, Th-232, and their decay products, along with K-40. Internal exposure from naturally occurring radionuclides is predominantly caused by U-238 and its daughter products, particularly Ra-226 and its subsequent decay products [4]. The distribution of these radionuclides is highly dependent on geological conditions and the type of rock present in the soil, resulting in varying concentrations across different locations [[5], [6], [7]]. Human activities such as mining, oil and gas extraction, and mineral processing can lead to elevated concentrations of these radionuclides [3,8]. The average worldwide activity concentrations of U-238, Th-232, and K-40 in soil are 33 Bq/kg, 45 Bq/kg, and 420 Bq/kg, respectively [3].

Mining activities pose a potential risk of exposure to NORMs in many African countries, including Ghana [3]. This risk arises from the lack of comprehensive regulations monitoring NORMs in ongoing mining sites, despite the presence of hundreds of active mines operating at various scales [3].

To address this issue, the International Atomic Energy Agency (IAEA) initiated a comprehensive radiological survey (from 2005 to 2007) under the Technical Cooperation (TC) Project GHA9005, titled “Development of Sustainable Radiation Protection and Waste Safety in Mining and Mineral Processing”. The major two mines in Ghana, AngloGold Ashanti in Obuasi and AngloGold Ashanti Iduapriem in Tarkwa, were investigated. The average activity concentrations of radionuclides, such as U-238, Th-232, and K-40, in soil were reported as 28.7 Bq/kg, 25.4 Bq/kg, and 581.8 Bq/kg for Obuasi and as 34.5 Bq/kg, 20.7 Bq/kg, and 682.4 Bq/kg for Tarkwa, respectively. Following the investigation, the average annual effective dose was estimated to be in the range of 0.28 ± 0.11 mSv and 0.29 ± 0.13 mSv for Obuasi and Tarkwa [9].

These findings could highlight the importance of adopting or establishing a set of instructions and regulations regarding NORM in mines and the need for comprehensive radiological surveys in other mining communities in Ghana. This would lead to a broader understanding of the presence of naturally occurring radioactivity in the mining and mineral processing sectors throughout the country, providing a valuable tool for achieving sustainable radiation protection and waste safety.

Following the completion of the TC project in 2007, the study on NORM was extended to the mining communities and environs, including oil and gas communities in Ghana. Several water bodies and water sources located within these communities and their environs serve as sources of drinking water, crop irrigation, and fishing. Hence, the study also included water samples from these communities, including water bodies polluted by illegal mining, popularly called “galamsey” (gather them and sell) in Ghana, and farmlands. This study aimed to measure, assess, and estimate some baseline radioactivity levels in these communities and, by extension, establish regional baseline data needed to make necessary recommendations to stakeholders in respect of public exposure.

The specific objectives of the study are:

• To quantify the radioactivity concentrations of U-238, Th-232, and K-40 in soils and Ra-226, Th-228 and K-40 water samples using gamma spectrometry.
• To estimate the radiological risk with respect to radiological hazard assessment, including external gamma dose rate, radium equivalent, external and internal hazard indices.
• To estimate the doses from the activity concentrations that the public is likely to receive.
• To assess the risk associated with the estimated doses.
• To determine the radiological suitability of soil in building materials.
• To provide recommendations on remedial measures if necessary.

Recently, the regulation of mining and mineral extraction industries concerning Naturally Occurring Radioactive Materials (NORM) waste has become a matter of great concern worldwide. Nations are required to evaluate the potential environmental implications of NORM wastes on a case-by-case basis, taking into account socio-political factors and cost reduction while minimizing exposure to the public and workers. Exemption Concentration Levels (ECL) for all radionuclides, including NORMs, have been established by national and international organizations such as the International Atomic Energy Agency (IAEA). Table 1 presents the exemption levels of the naturally occurring radionuclides, established based on the following principles [10,11]:

a. That the radiation risks to individuals as a result of exempted practices or sources are of no regulatory concern;
b. Be under the prevailing circumstances, it is required that the collective radiological impact of the exempted practice or source is sufficiently low so as not to warrant regulatory control, and
c. That the exempted practices and sources are inherently safe, with no significant likelihood of scenario failures to satisfy principles (a) and (b) above.

**Table 1:** Exempt concentration levels in Bq/kg naturally occurring radionuclides.

Naturally occurring Radionuclide	Ra-226	U-238
Exemption Concentration (Bqkg^−1^)	1000	1000

The activity concentrations in the extractive media are required to comply with the levels of the exempt activity concentrations. In situations where the levels of Naturally Occurring Radioactive Materials (NORM) are significantly high, industrial processes will need to be modified to ensure compliance with recommended levels. This is necessary to address concerns regarding the NORM waste generated during regular activities. As a result, the initial licensing processes for mineral exploration should incorporate estimates of the volume of NORM waste to be generated. This includes the concentration of natural radionuclides, such as U-238, Th-232, and K-40, and the techniques to be employed for the treatment and management of such waste. The cost of mineral exploration should also take into account the production, treatment, and management of NORM waste.

This study seeks to compile data on the radioactivity concentrations of U-238, Th-232, and K-40 in soil and Ra-226, Th-228, and K-40 in water within mining communities. This will provide a general overview with the overall aim of establishing regional data on NORM in Ghana.

## 2 Materials and methods

### 2.1 Study areas

**Fig. 1:**
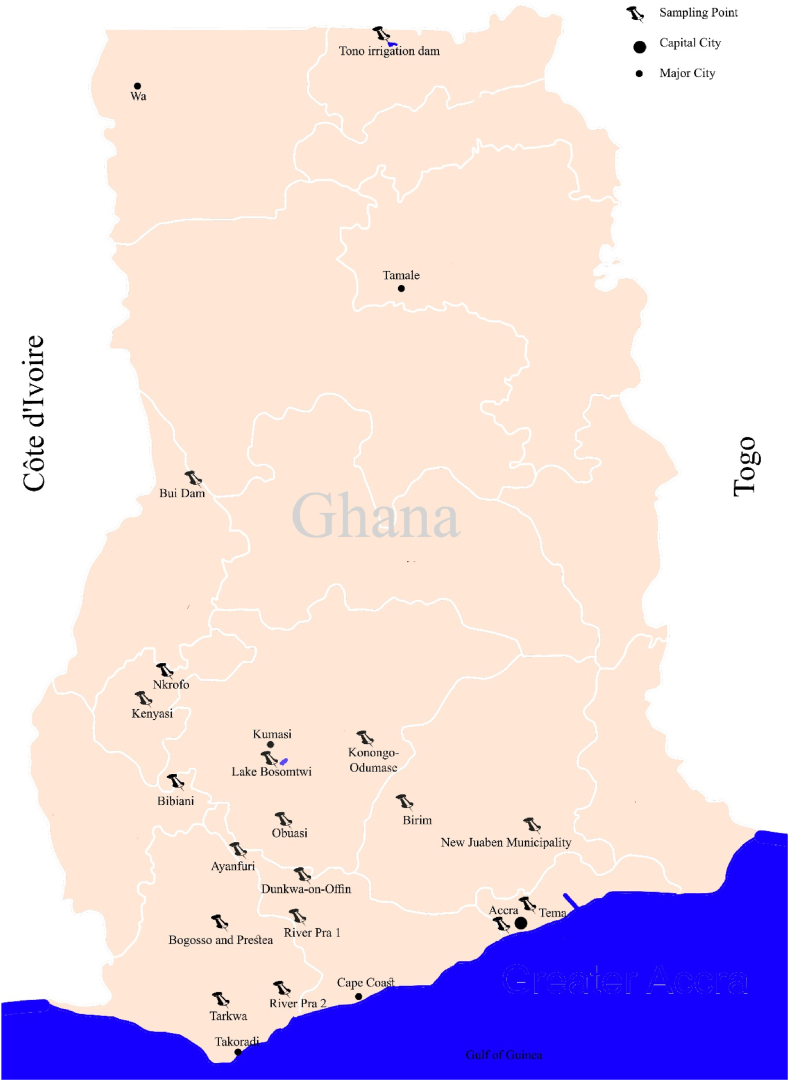
Locations of samples and their distribution.

The study areas are described by regions, as illustrated in Fig. 1, within which the levels of Naturally Occurring Radioactive Materials (NORMs) were assessed. Detailed information about the sampling points, types of samples, and the number of samples collected from each site is presented in Table 2.

**Table 2:** Regional representation of sampling study area and samples.

Regions	Study area	Samples Taken	Number of Samples
Ashanti	Obuasi	Soil and water	Soil (10), water (5)
	Konongo-Odumase	Soil and water	Soil (8), water (3)
	Lake Bosomtwi	Sediment and water	Sediment (8), water (3)
Brong Ahafo	Kenyasi	Soil and water	Soil (8), water (3)
	Bui Dam	Sediment and Water	Sediment (8), water (3)
Central	Ayanfuri	Soil and water	Soil (10), water (5)
	Dunkwa-on-Offin	Soil and water	Soil (8), water (3)
	River Pra 1	Sediment and water	Sediment (10), water (5)
Eastern	Birim	Soil and water	Soil (10), water (5)
	Farmlands in New Juaben Municipality	Soil and water	Soil (8), water (3)
Greater Accra	Tema	Soil and water	Soil (8), water (3)
	Accra (Beach Sand Along the Coastal Belt of the Greater Accra Region	Sand	Sand (10)
Western	Tarkwa	Soil, water, sludges, and scales	Soil (10), water (5), Sludge (5), scales (5)
	Bibiani	Soil and water	Soil (8), water (3)
	Nkrofo	Soil and water	Soil (8), water (3)
	Bogosso and Prestea	Soil and water	Soil (8), water (3)
	River Pra 2	Sediment and water	Soil (10), water (5)
Upper East	Tono irrigation dam	Sediment and water	Sediment (8), water (3)

#### 2.1.1 Ashanti region

##### 2.1.1.1 Obuasi and its environs

The study area is the gold mining town of Obuasi, located in the Adansi West District of the Ashanti Region of Ghana. Obuasi is situated at latitude 06°12′ North of the equator and longitude 01°40′ West of the Greenwich Meridian, approximately 200 km north of the Coast of the Gulf of Guinea [12]. It covers a total land area of 162.4 km^2^ and constitutes about 17% of the Adansi West District, which has a total area of 950 km^2^. According to the 2003 Environmental Impact Assessment report [12], the current population of Obuasi is 115,564, making it the largest town in the Adansi West District. The area exhibits an average population density of about 712 persons per square kilometre and is experiencing a rapid annual growth rate of 4%, making it one of the fastest-growing areas in the country. This significant increase in population is attributed to the expansion of mining activities in the area, leading to increased production levels. These developments incentivize people, particularly from rural areas, to migrate to Obuasi seeking opportunities in the mining sector. Many of these migrants are young individuals in search of employment opportunities in mining operations.

##### 2.1.1.2 Konongo-Odumase and its environs

The study area is located at latitude 6°37′00″ N and longitude 1°13′00″ W, approximately 53 km from the Ashanti Regional Capital, Kumasi, and about 200 km from the national capital. It has a population of about 41,238 and is the capital of the Asante Akyem Central District. Konongo is known for its historical significance in gold and manganese mining. The topography of the area is characterized by undulating landscapes, and it experiences tropical rainfall for most parts of the year.

The inhabitants primarily rely on boreholes and hand-dug wells as their main sources of water, along with some surface water sources [13]. These boreholes and hand-dug wells are influenced by the development of porosity through cleavage and weathering. Weathering depths are greatest in the Birimian and Tarkwaian systems, attributable to the limited primary porosity nature of the consolidated rock formations in these systems [13].

##### 2.1.1.3 Lake Bosomtwe, Kumasi

Located in the Ashanti Region, Lake Bosomtwi is a pristine freshwater lake situated approximately 30 km southeast of Kumasi. Nestled within the forested area at the northern end of the Adansi mountains, it offers a tranquil and picturesque setting. The lake boasts a radial discharge of 106 km^2^, with a maximum width of about 11 km at its widest points, a depth reaching up to 78 m, and covering an area of about 52 km^2^ [14]. Despite its lack of a natural outlet, Lake Bosomtwi has experienced significant overflow in recent times [15]. The primary regulation of the lake’s water system is through precipitation, occurring directly as rainfall into the lake and evaporation directly from the lake’s surface [15]. Additionally, there is a secondary, albeit insignificant, runoff from the surrounding watershed into the lake. Considering hydrogeological conditions, it is reasonable to assume that little or no groundwater enters or leaves the basin. The lake's sensitivity to even minor variations in precipitation [14,15] and climate-related factors, including mean annual temperature and evaporation rates [16], is well recognized.

Lake Bosomtwi holds immense significance for the 24 surrounding communities, serving as their primary source of sustenance. The local residents heavily rely on fishing as a means of livelihood, providing both income and a protein-rich food source. Furthermore, the lake is a crucial source of drinking water and irrigation for agricultural activities. Its presence also facilitates transportation and ecotourism opportunities, contributing significantly to the social and economic development of the region.

#### 2.1.2 Brong Ahafo region

##### 2.1.2.1 Kenyasi and its environs

Kenyasi is the capital of the Asutifi North district in the Brong Ahafo Region. It is located at latitude 6°58′60″ N and longitude 2°22′60″ W, with a population of about 15,637 [[17], [18], [19]]. Situated approximately 300 km northwest of Accra and 50 km from Sunyani [19], the inhabitants of Kenyasi are primarily engaged in cash crop farming, in addition to mining activities. Major crops grown in this area include cocoa, plantain, cassava, and coffee [18].

Geologically, the study area possesses natural resources or potential for granite, clay, sand, gold, and diamond deposits in the Birimian and Dahomeyan formations [17]. The Birimian Formation is particularly known for containing gold deposits associated with mixed (meta)spheroidal sedimentary rocks and (meta)magmatic volcanic units in the footwall of the Kenyasi Thrust Fault [17,20]. These rocks exhibit considerable potential for the presence of manganese and bauxite. Currently, gold mining is underway in the area, primarily conducted through open pit methods, with a few instances of underground mining [20]. Studies have also indicated the abundance of diamonds in the study area, with ongoing exploration activities [17]. Additionally, there is an extensive deposit of sand and clay within the environs [17].

##### 2.1.2.2 Bui dam

The Bui Dam, initially constructed as part of a 400-MW (540,000 hp) hydroelectric power project [21,22], also serves as a source of water for crop irrigation and fishing. It is situated on the border of the Northern Region and the Brong-Ahafo Region of Ghana, at coordinates 8°16′42″N 2°14′9″W. Located at the southern end of the Bui National Park at Bui Gorge, the dam is built on the Black Volta River.

The Bui Dam stands at a height of 108 m and stretches 492 m in length, with an elevation at the crest of 185 m [21]. It boasts a total capacity of 12.57 billion cubic meters, with surface areas of 288 km^2^ and 444 km^2^ at depths of up to 88 m [22].

#### 2.1.3 Central region

##### 2.1.3.1 Ayanfuri and its environs

Ayanfuri is a mining town located in the upper Denkyira west district of the Central Region of Ghana, approximately 75 km west of Accra by road. Positioned at latitude 5°28′37.8″ N and longitude 0°52′03.5″ W, it has a population of about 4660. The residents of Ayanfuri are largely engaged in mining and crop farming. The local geology consists of Ashanti-type clastic sediments, granite plugs, and dykes along two or three regional shear structures. In the Ayanfuri locality, there are more than twenty-four gold deposits, most of which are hosted by granite intrusives, accounting for more than 80% of the known gold reserves [6,23].

The later deposits in the area were predominantly formed in a ductile system, characterized by discontinuous, pinched-out, and bulging gold veins of higher grade. In contrast, the deposits in the granite were developed in friable rock, resulting in significantly larger deposits with more evenly distributed, though lower grade, gold content. Most of the known gold resources in Ayanfuri are derived from granite caps and sills or dykes along the same structures that intersect the sediments containing the gold deposits.

Gold in this study area is associated with less than 3% pyrite, minor arsenopyrite, and traces of galena, chalcopyrite, rutile, and sphalerite [23]. The gold is present in the form of exceptionally fine grains, often found along the boundaries of sulphide grains and within sulphide fractures. It is typically located at or near vein boundaries, occasionally exhibiting coarse grains visible within the quartz.

The geological composition of the area includes metamorphic rock, featuring intrusive igneous rocks containing minerals such as arsenopyrite, pyrite, quartz, and feldspar, among others [23].

##### 2.1.3.2 Dunkwa-on-Offin

Dunkwa-on-Offin, located at latitude 5°58′3.3″ N and longitude 1°47′15.3″ W, is the capital of the Upper Denkyira East district in the South-Central Region of Ghana. According to the 2013 census, it has a population of 33,379 [[24], [25], [26]]. This study area was chosen due to the high incidence of small-scale mining and ‘galamsey’ activities, characterized by abandoned mine shafts, prevalent gravel piles, and, in some cases, clogged watercourses [26].

The main soils in the area are forest ochrosols, varying in color from brown to orange. These soils are considered some of the best in the country for crop production, as they are nutrient-rich and alkaline in nature, attributed to the decrease in rainfall within the area. Cash crops such as cocoa, oil palm, cassava, maize, and banana thrive here, covering about 50% of the total cultivable study area [25].

Geologically, the settlement consists mainly of the Birimian and Tarkwa Formations, which include metamorphosed sediments such as phyllites, schists, and lavas [26]. This geological composition explains the rich mineral deposits in the area, particularly the alluvial gold deposits in the valleys of the Offin River and its tributaries, as well as interior gold deposits [26,27]. The inhabitants of Dunkwa-on-Offin are predominantly miners and farmers.

##### 2.1.3.3 River Pra

The Pra River basin, a part of Ghana’s southwestern hydrographic system, encompasses a drainage area of 23,188 km^2^ and has an average annual flow of 214 m³/s, traversing four regions: Central, Western, Ashanti, and Eastern [28]. The climate in the basin is relatively humid, with relative humidity ranging from 60 to 95% and an annual rainfall of 1500–2000 mm. During the rainy season, the basin is significantly influenced by the humid southwest monsoon. Geologically, the rocks underlying the region are of Precambrian age and are predominantly classified as Birimian and Tarkwaian, while the vegetation consists of moist semi-deciduous forests accompanied by ochrosols, which are alkaline soils [28].

The study area was selected due to the elevated levels of pollution in the river and the prevalence of illegal mining activities within its vicinity. These mining practices, coupled with the climatic conditions, can increase the concentration of natural radionuclide activities in the environment through processes such as runoff and leaching [28]. Soil sediments were sampled from areas where the water is contaminated by ‘galamsey’ activity or gold ore processing in the river, particularly in the heavily affected Central, Western, and Eastern regions.

#### 2.1.4 Eastern region

##### 2.1.4.1 New Abirim

New Abirim, located at latitude 6°20′40″ N and longitude 0°59′53″ W, is situated 138 km west of Koforidua, the Eastern regional capital, and 179 km northwest of Accra. With a population of about 10,000, it serves as the district capital of the Birim North District [29,30]. The area is dotted with several hamlets and farmsteads, reflecting the fact that the majority of the residents are skilled in crop farming and pisciculture [31]. Benefitting from a favorable rainfall pattern and fertile lands, the major crops farmed in this area include plantain, citrus, cocoa, rice, oil palm, cassava, maize, and vegetables [32].

The local geology of New Abirim is characterized by deposits situated along a regional fault that trends parallel to the regional structures, with foliation developed in a Birimian host rock [[33], [34], [35]]. This geological setting is conducive to gold mining, which is a significant activity in the area.

##### 2.1.4.2 Farmlands in New Juaben municipality

The New Juaben Municipality, situated at latitude 6°13′14″ N and longitude 0°31′84″ W, has a population of 183,727 and is one of the administrative districts in the Eastern region. It encompasses fifty-two (52) communities and covers a land area of 159 km^2^ [36,37]. Characterized by a semi-deciduous rainforest climatic zone, the region boasts rich, fertile farmlands that are particularly well-suited for small to medium-scale crop farming, as well as cattle rearing and poultry farming [25,36].

The Densu River Basin and its tributaries, which include dammed sections, serve as the main source of water for the farmlands and industrial processes in the area. Additionally, these water bodies provide for human and animal consumption, playing a crucial role in the livelihoods and sustenance of the local population.

#### 2.1.5 Greater Accra region

##### 2.1.5.1 Tema

Tema, the main industrial city in Ghana, has a population of 292,773 and is uniquely located at the intersection of the equator and the Greenwich Meridian on the coast of West Africa [38]. The city is home to approximately thirty (30) major industrial companies. Among these is an oil refinery, initially incorporated in 1960 as the Ghanaian Italian Petroleum Company Limited, as well as the country’s primary harbour.

The sludge, scales, and other waste materials, particularly from the refinery, have the potential to accumulate Naturally Occurring Radioactive Materials (NORMs) at significant levels. This accumulation could pose a potential source of radiological risk to both workers and inhabitants of the community [39].

#### 2.1.6 Beach sand along the coastal belt of Greater Accra Region

Greater Accra, the capital of Ghana and a hub for most of the country's economic activities, encompasses thirty-two (32) districts and covers a land area of 3245 km^2^. With a population of 5,455,692, it accounts for 17.7% of Ghana's total population [40]. The region boasts a coastline that stretches for about 125 km between the boundaries of the Central Region and the Volta Region, featuring numerous beach resort locations that attract holidaymakers and tourists. Additionally, inhabitants along the coastline are engaged in the production of beach sand bricks, contributing to the local economy.

#### 2.1.7 Western region

##### 2.1.7.1 Tarkwa

Tarkwa, situated at latitude 5° 15′ N and longitude 2° 00′ W, is approximately 300 km west of Accra, Ghana’s capital. The local population is primarily involved in subsistence farming, but gold mining is the main industrial activity within the study area [41]. Tarkwa lies within Ghana's main gold belt, the Tarkwaian system, which is a part of the larger Ashanti Belt that extends between Axim and Konongo [[42], [43], [44], [45]]. The local geology is characterized by the dominance of the Kawere Group, the Banket series, Tarkwa Phyllite, and Huni Sandstone, all of which have significant gold mineralization content [23,42,44].

Tarkwa is also recognized for offshore oil and gas exploration and production activities. Waste materials such as scales and sludges are commonly shipped onshore for storage. Therefore, this area was chosen to assess the radiological impact of Naturally Occurring Radioactive Materials (NORMs) in scales and sludges. The results of this assessment will assist decision-makers and stakeholders in recommending the most effective methods for oil and gas companies to manage residues concentrated with NORMs.

##### 2.1.7.2 Bibiani

Bibiani, a gold mining town and the capital of the Bibiani-Anhwiaso-Bekwai district, is located at latitude 6°27′10.6″ N and longitude 2°18′53.85″ W, approximately 253 km northwest of Accra. The town has a population of 21,583 [46,47]. Mining practices in the area are primarily conducted through open pit methods, complemented by a few underground mining operations [46,47]. In addition to gold mining, the inhabitants of Bibiani largely rely on subsistence crop farming.

Geologically, the occurrence of gold mineralization in this area can be attributed to significant hydrothermal alteration, predominantly characterized by ankerite-albite-muscovite alteration. This type of alteration is present in both the Suraw sub-basin of the Tano Basin and the main Ankobra Basin [46,47].

##### 2.1.7.3 Nkroful and its environs

Nkroful, a mining community with 36,409 inhabitants, constitutes 1.5% of the region's population and is the capital of the Nzema East Municipality of the Ellembelle District. It is located at latitude 4°57′50″ N and longitude 2°19′20″ W, approximately 5 km from the Gulf of Guinea and 76 km west of Takoradi, the capital of the Western Region [36]. The town is endowed with natural resources that support agricultural activities, including coconut palm, oil palm, and cocoa farming. Additionally, gold mining is a significant activity within the study area.

The gold deposits in Nkroful are predominantly found within the Proterozoic Birimian formation. This formation is characterized by NE-SW striking gold belts that occur along the boundaries of metavolcanic belts and metasedimentary basins [25]. These gold deposits are closely associated with sulphide minerals, primarily arsenopyrite, pyrite, and pyrrhotite, while sphalerite and tetrahedrite are present to a lesser extent.

##### 2.1.7.4 Bogoso and Prestea environs

Bogoso and Prestea are mining towns within the Prestea-Huni Valley district in the Western Region of Ghana, with populations of about 36,409 and 35,760 respectively [48]. Bogoso, located at latitude 5°33′57″ N and longitude 2°00′58″ W, is situated about 78 km north-northwest of Takoradi, the capital of the Western Region, and 202 km west of Accra, the capital of Ghana. It is approximately 21 km from Prestea. Prestea, positioned at latitude 5°25′58″ N and longitude 2°08′34″ W, lies about 72 km northwest of Takoradi and approximately 217 km west of Accra.

The topography of the area is characterized by a series of ridges running southwest to northeast, manifested as low, steep-sided hills with broad valleys in between. These valleys contain seasonal streams. The gold reservoirs in these towns are primarily located on the western slopes of the ridges. There is extensive oxidation of the upper layers of the reservoirs, which can be several tens of meters deep in some places. Generally, oxidation is deeper in areas of greater relief and shallower in the valleys and regions with earlier mining activity [25,49].

Subsistence agriculture is widespread throughout the area, with the main crops being plantains, pineapples, coconuts, cassava, maize, yams, coffee, and some oil palms [25,49]. Gold mining has been a commercial activity in these areas since the early 20th century [49].

#### 2.1.8 Upper East region

##### 2.1.8.1 The Tono irrigation dam

The Tono irrigation dam, constructed on the Tono River and covering a total area of 3600 ha, is located in the Kassena Nankana East district of the Upper East region. It serves nine (9) villages with a combined population of about 5000. The district is situated between latitudes 10.45° N and 10.90° N, and longitudes 1.00° W and 1.30° W. The prevailing climate is mostly Sahelian (hot and dry), characterized by semi-arid grassland interspersed with short trees. This region experiences two main climatic seasons: the wet and the dry seasons. The wet season supports crop farming, while the irrigation dam becomes a crucial resource during the dry season, supplementing living and farming needs for both urban and rural communities. The dam is a major source of water for domestic purposes, including bathing and washing [[50], [51], [52]].

Fish harvested from the dam are widely patronized within the Upper East region. Additionally, the water from the dam is utilized by livestock and for various domestic purposes [50,51]. Some inhabitants also use sediments from the dam in molding bricks for the construction of their homes [52]. Overall, the dam plays a significant role in maintaining food production in the area.

### 2.2 Sampling and sample preparation

In each of the selected study areas, soil samples were systematically collected, with five replicates obtained from each location. The soil specimens then underwent a meticulous preparation process, beginning with initial air-drying at room temperature. Following this, the samples were subjected to controlled drying in an oven for 12 h at a constant temperature of 105 °C, until a stable weight was achieved.

To ensure uniformity, the dried soil samples were finely ground and sieved through a mesh with a pore size of 2 mm. For the water samples, a precise volume of 1.5 L was drawn from each sampling area and securely stored in plastic containers. To prevent the potential adhesion of radionuclides to the container walls, two drops of hydrochloric acid were added to each water sample, preserving the integrity of the radionuclide content within the water matrix.

Following these preparatory steps, the homogenized samples were meticulously sealed within aluminum Marinelli beakers. An essential part of the protocol involved allowing these sealed samples to equilibrate for 28 days before measurement commencement. This incubation period is crucial to achieve equilibrium between radium and its short-lived progeny, as indicated in previous studies.

To maintain rigorous quality assurance throughout the sampling process, ‘Standard Method for Sampling Surface Soils for Radionuclides' and ‘Recommended Practice for Investigation and Sampling Soil and Rock for Engineering Purposes' were adhered to at each sampling point, along with an internal QA guidebook for sample preparation [53,54]. The type of sample taken at each study area has been introduced previously in Table 2.

### 2.3 Instrumentation, calibration and measurments

For the measurement of radionuclides present in the samples, a gamma spectrometry system was employed, incorporating an n-type High-Purity Germanium (HPGE) detector and a computer-based multi-channel analyzer (MCA). The detector's relative efficiency is 40%, with an energy resolution of 1.8 keV at the 1332 keV gamma-ray energy of Cobalt-60 (60Co). The identification and quantitative analysis of each radionuclide were conducted using their gamma-ray energies and spectrum via the Genie 2000 software.

To minimize background radiation, the detector was shielded with 100 mm of lead (Pb), lined with layers of copper, cadmium, and plexiglass (3 mm each). Additionally, the detector was cooled using liquid nitrogen to maintain a temperature of −196 °C. For background correction, ten empty 1-L Marinelli beakers filled with distilled water were counted for 36,000 s to determine the environmental background distribution around the detector.

A standard radionuclide source, NW146, manufactured by QSA Global GmbH, with a volume of 1000 ml and a density of 1.0 g/cm³, and known activities, was used to determine the peaks' counting efficiency.

### 2.4 Activity concentrations

The activity concentration in the samples was determined by identifying the specific energy peaks of related radionuclides: Bismuth-214 (Bi-214) at 609.31 keV for Radium-226 (Ra-226), Actinium-228 (Ac-228) at 911.21 keV for Thorium-232/228 (Th-232/Th-228), and Potassium-40 (K-40) at 1460.83 keV. This was achieved using Equation (1).

(1) $$A_{\left(s,w\right)}=\frac{N}{\rho \times T\times \varepsilon \times m}$$

where,

N is the net count of the radionuclide in the samples,

ρ is the gamma-ray yield,

ε is the counting efficiency,

T is the counting time,

m is the sample mass (kg) or volume (l),

A is the activity concentrations in Bq/kg for soil and Bq/L for water.

### 2.5 External gamma dose rate

The calculation of the external gamma dose rate (Dγ) at a height of 1.0 m above the ground for the soil samples was performed using their respective activity concentrations, as outlined in Equation (2) [7].

(2) $$D_{\gamma }\left(nGy/h\right)=A_{U}\times {DCF}_{U}+A_{Th}\times {DCF}_{Th}+A_{K}\times {DCF}_{K}$$

where, ${DCF}_{U}=0.462,{DCF}_{Th}=0.604\;\text{and}\;{DCF}_{K}=0.0417$ are the conversion factor for, U-238, Th-232, and K-40, respectively in nGy/h per Bq/kg [3,9,25] and, $A_{U},A_{Th}\;\text{and}\;A_{K}$ are the activity concentration of each radionuclide.

### 2.6 External and committed effective doses

The average annual effective doses (Eɣ), expressed in millisieverts per year (mSv/y), were calculated by applying the absorbed dose rate. This calculation utilized the dose conversion factor of 0.7, along with an outdoor occupancy factor of 0.2, as specified in Equation (3).

(3) $$E_{\gamma }\left(mSv/y\right)=D_{\gamma }\times 8760\times 0.2\times 0.7$$

The annual committed effective doses (E_ing_) from water consuming, 730 L/year, were estimated using Equation (4) to calculate.

(4) $$E_{ing}\left(mSv/y\right)=I_{w}\sum {DCF}_{ing}\left(Ra,Th,K\;\right)A_{w}$$

where, A_w_ is the activity concentration in Bq/L, I_w_ is the consumption rate of water and DCF_ing_ are the ingestion dose coefficient for Ra-226 = 2.8 × 10^−4^ mSv/Bq, for Th-228 = 7.2 × 10^−5^ mSv/Bq and, for K-40 = 6.2 × 10^−6^ mSv/Bq [3,10,55].

The total annual effective dose (ET) was calculated using Equation (5) based on the ICRP dose calculation method [1,2].

(5) $$E_{T}=E_{\gamma }+E_{ing}$$

### 2.7 Radium equivalent, radiological hazard indices and excess lifetime cancer risk

In light of the radiological health impact of radon and its short-lived daughters, the radium equivalent activity, as well as the external and internal hazard indices, were evaluated to assess the potential hazard associated with using soils or NORM residues as building materials in these mining communities. The utilization of soils and NORM residues as building materials is quite common among the inhabitants of these areas.

The corresponding radium equivalent activity (Ra_eq_), external hazard index (H_ex_), and internal hazard index (H_in_) were calculated using Equations (6), (7), (8), respectively. In these equations, C_Ra_, C_Th_, and C_K_ represent the respective activity concentrations of Ra-226, Th-232, and K-40 [5,56,57,58].

(6) $$Ra_{eq}=C_{Ra}+1.43C_{Th}+0.077C_{K}$$

(7) $$H_{ex}=\frac{C_{Ra}}{370}+\frac{C_{Th}}{259}+\frac{C_{K}}{4810}$$

(8) $$H_{in}=\frac{C_{Ra}}{185}+\frac{C_{Th}}{259}+\frac{C_{K}}{4810}$$

The radium equivalent in any potential building materials such as soils must be less than 370 Bq/kg while that of the and the external and internal hazard indices must be less than one to be recommended as safe for use; indicative that, the NORM radiation exposure from construction materials must be less than 1.5 mSv/y [56,59].

Also, to ascertain the likelihood of the public risk to cancer over time, due to these exposures, the Excess Lifetime Cancer Risk (ELCR) were estimated using the following Equation (9), where E, is either the external or internal the annual effective dose equivalent, DL, the average life expectancy estimated to be 70 years [1,3] and RF, the risk factor for stochastic effects, recommended by ICRP 60 for the public to 0.05 Sv, [1,3].

(9) ELCR=E×DL×RF

The global recommended value of ELCR is given as 0.29 × 10^−3^ for external exposures and 1.16 × 10^−3^ for internal exposures [3].

## 3 Results and discussion

Table 3 presents the average activity concentrations of U-238, Th-232, and K-40 in soil samples. The average value for U-238 activity concentration is 59 ± 16 Bq/kg, ranging from 5 to 730 Bq/kg. For Th-232, the average activity concentration is 48 ± 15 Bq/kg, within a range of 5–460 Bq/kg. The activity concentration for K-40 averages at 286 ± 57 Bq/kg, ranging from 2 to 1168 Bq/kg.

**Table 3:** Activity concentration of U-238, Th-232, and K-40 in soil samples.

Region	Study area	Average and Range of Activity Concentration, Bqkg^−1^		
		U-238	Th-232	K-40
Ashanti	Obuasi	43 ± 8 (29–52)	33 ± 6 (25–44)	632 ± 142 (582–736)
	Konongo-Odumase	9 ± 1 (2–17)	22 ± 1 (2–37)	200 ± 4 (39–407)
	Lake Bosomtwi (Sediment)	8 ± 1 (7–10)	6 ± 1 (5–8)	276 ± 22 (226–304)
Brong Ahafo	Kenyasi	15 ± 6 (8–26)	27 ± 8 (9–67)	157 ± 16 (60–249)
	Bui Dam (sediment)	5 ± 1 (2–10)	5 ± 1 (3–7)	129 ± 24 (98–133)
Central	Ayanfuri	65 ± 2 (29–97)	72 ± 2 (35–117)	1168 ± 68 (500–1796)
	Dunkwa-on-Offin	25 ± 1 (11–45)	29 ± 6 (10–68)	226 ± 94 (96–409)
Central, Western and Eastern	River Pra (sediment)	25 ± 2 (8–42)	28 ± 6 (4–55)	233 ± 67 (85–479)
Eastern	New Abirim	12 ± 1 (5–21)	11 ± 2 (3–24)	140 ± 25 (24–426)
	Farmlands in New Juaben Municipality	13 ± 3 (6–18)	11 ± 3 (6–17)	206 ± 30 (87–344)
Greater Accra	Tema Metropolis	7 ± 1 (5–9) 11±1^a^ (10–32)	17 ± 3 (13–16) 33±3^a^ (17–231)	62 ± 9 (78–110) 95 ± 19^a^ (68–184)
	Beach sand along the coastal belt of the Greater Accra Region	20 ± 1 (11–32)	43 ± 4 (17–231)	110 ± 20 (68–184)
Western	Tarkwa	42 ± 4 (35–49) 730 ± 241^b^ (557–898) 37± 1^c^ (36–38)	30 ± 5 (21–37) 460 ± 226^b^ (297–617) 30± 1^c^ (29–30)	682 ± 134 (618–694) 48±3^b^ (38–57) 2± 1^c^ (2–3)
	Bibiani	10 ± 5 (3–25)	9 ± 7 (1–26)	237 ± 144 (24–580)
	Nkrofo	8 ± 1 (7–16)	44 ± 1 (13–87)	395 ± 123 (284–508)
	Bogosso-Prestea	87 ± 8 (4–235)	39 ± 2 (27–51)	345 ± 91 (320–360)
Upper East	Tono Irrigation dam (sediment)	7 ± 1 (5–9)	7 ± 1 (6–8)	380 ± 95 (219–453)
Average ± σ		**59 ± 16**	**48 ± 15**	**286 ± 57**
Range		**5–730**	**5–460**	**2–1168**
World average [3]		**33**	**45**	**420**

σ—Standard deviation.

a Soil from oil refinery site.

b Oil & gas Sludge.

c Oil & gas Scales.

The results from Table 3 indicate that the highest activity concentrations of U-238 and Th-232 were recorded in sludge samples, suggesting that these constitute the major NORMs in all the samples under study. Apart from the sludge samples, significant activity levels of NORMs were also recorded in Obuasi and Ayanfuri. The overall average concentrations of U-238 and Th-232 are approximately twice as high as the world average values. However, the average activity concentration of K-40 was found to be lower.

Although the average activity levels of U-238 and Th-232 in this study are higher than the worldwide average values, they are still well below the exemption values of 1000 Bq/kg for U-238 and Th-232 (Table 1), which would warrant regulatory control [3,10,11,55]. Nevertheless, there is a need for considerable regulation of NORMs within mining areas, as the average annual effective dose from terrestrial gamma rays exceeds the world average.

Following Table 4 data, the descriptive statistics for the radioactive elements across the regions are as follows:

**Table 4:** The descriptive statistics for the radioactive elements across the regions in soil samples.

Statistic	U-238 (Bq/kg)	Th-232 (Bq/kg)	K-40 (Bq/kg)
Count	17	17	17
Mean	23.59	25.12	328.12
Std Dev	23.14	17.93	275.56
Min	5	5	62
25%	8	11	157
50% (Median)	13	27	233
75%	25	33	380
Max	87	72	1168
Range	82	67	1106
Variance	535.51	321.61	75935.11
IQR	17	22	223

Count: Number of observations. Mean: Average value. Std Dev: Standard Deviation, a measure of the amount of variation or dispersion in the data. Min: Minimum value. 25%: First quartile. 50% (Median): The median, a better measure of central tendency for skewed data. 75%: Third quartile. Max: Maximum value. Range: Difference between the max and min values. Variance: Measure of the data's spread. IQR: Interquartile Range, measuring the middle 50% of the data.

The results of the ANOVA tests for each element across different regions shows in Table 5.

**Table 5:** The ANOVA tests for each radionuclide in soil across different region.

	U-238	Th-232	K-40
F-Value	0.85	1.03	0.89
P-Value	0.58	0.48	0.56

In all cases, the p-values are greater than the typical alpha level of 0.05, suggesting that there are no statistically significant differences in the levels of U-238, Th-232, and K-40 across the different regions.

Inferential statistical analysis, including regression, was conducted to explore relationships and infer patterns from the datasets for the relationship between the activity concentrations of Uranium-238, Thorium-232, and Potassium-40 in soil samples. To perform a regression analysis with dataset, a common approach using one element's concentration to predict another's were used in this analysis. Based on the nature of the data, multiple linear regression models were applied which can provide insights into how these elements' concentrations are interrelated.:

*Predicting U-238 concentration based on Th-232 and K-40 concentrations.*

*Predicting Th-232 concentration based on U-238 and K-40 concentrations.*

*Predicting K-40 concentration based on U-238 and Th-232 concentrations.*

The regression analysis results for each model are as follows:

*Predicting U-238 concentration based on Th-232 and K-40 concentrations*:

R-squared: 0.806 (This indicates that 80.6% of the variability in U-238 concentration can be explained by Th-232 and K-40 concentrations.).

The coefficients suggest that both Th-232 and K-40 concentrations are positively correlated with U-238 concentration. However, only the coefficient for K-40 is statistically significant (P < 0.05).

*Predicting Th-232 concentration based on U-238 and K-40 concentrations:*

R-squared: 0.769 (This indicates that 76.9% of the variability in Th-232 concentration can be explained by U-238 and K-40 concentrations.).

The coefficients suggest that both U-238 and K-40 concentrations are positively correlated with Th-232 concentration, with U-238 showing a statistically significant coefficient (P < 0.05).

*Predicting K-40 concentration based on U-238 and Th-232 concentrations:*

R-squared: 0.769 (Similarly, 76.9% of the variability in K-40 concentration can be explained by U-238 and Th-232 concentrations.).

The model indicates a positive correlation between K-40 concentration and both U-238 and Th-232 concentrations, but only the coefficient for U-238 is statistically significant (P < 0.05).

These results suggest that there are significant relationships between the concentrations of these radioactive elements in the soil samples. The relatively high R-squared values in all models indicate that a significant proportion of the variability in each element's concentration can be explained by the concentrations of the other two elements. However, it is important to interpret these results cautiously, as the underlying causative mechanisms might be complex and beyond the scope of a simple linear regression analysis.

**Fig. 2:**
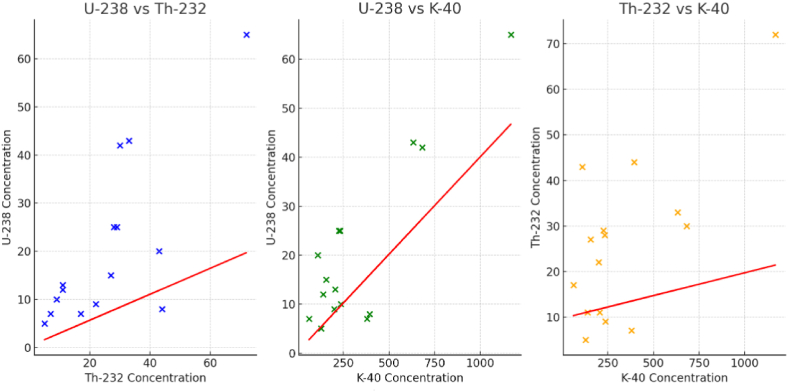
Scatter plot of radionuclides concentrations with the regression lines in soil samples.

**Fig. 3:**
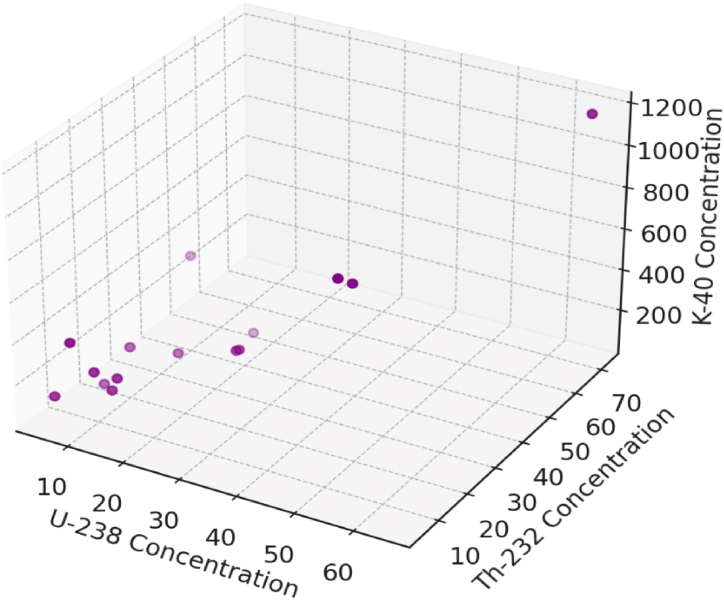
3D scatter plot to visualize the relationships among radionuclides in soil samples.

For each pair of radionuclides, scatter plots with the regression lines were created and shows in Fig. 2, while Fig. 3 shows the concentrations of U-238, Th-232, and K-40 in a three-dimensional space each point represents a sample with its respective concentrations for the three radionuclides, which provides a comprehensive view of how these elements' concentrations are interrelated in the dataset.

Table 6 presents the results of the radiological hazard assessment, including the radium equivalent activity, the external hazard index, the internal hazard index of the soil samples, and the estimated annual effective doses. This assessment was conducted to evaluate whether the soil samples from the study areas could pose a risk of public radiation exposure if used as building materials. Typically, the assessment of radiological hazards in building materials involves analyzing the activity concentrations of Ra-226, Th-232, and K-40. Ra-226 is used as a surrogate for U-238 since approximately 98.5% of the radiological hazard from the uranium series originates from radium and its decay products, particularly radon [60].

**Table 6:** Average absorbed dose rates, radium equivalent activities, external and internal hazard indices, and annual effective doses of U-238, Th-232 and K-40 in soil samples.

Study area	Region	Average Absorbed dose rate, (nGy/h)	Radium equivalent activity, (Bq/kg)	External hazard index	Internal hazard index	Average annual effective dose, (mSv/y)	Excess Lifetime Cancer Risk (ELCR) 10^–3^
Ashanti	Obuasi	67 ± 13	139 ± 28	0.37 ± 0.07	0.49 ± 0.09	0.08 ± 0.02	0.28
	Konongo-Odumase	26 ± 1	55 ± 3	0.15 ± 0.01	0.17 ± 0.01	0.03 ± 0.01	0.11
	Lake Bosomtwi	19 ± 1	38 ± 2	0.10 ± 0.01	0.12 ± 0.01	0.02 ± 0.01	0.07
Brong Ahafo	Kenyasi	30 ± 10	66 ± 19	0.18 ± 0.05	0.21 ± 0.09	0.04 ± 0.02	0.14
	Bui Dam (sediment)	11 ± 2	22 ± 5	0.06 ± 0.01	0.07 ± 0.02	0.02 ± 0.01	0.07
Central	Ayanfuri	742 ± 260	258 ± 16	0.70 ± 0.04	0.87 ± 0.05	0.15 ± 0.01	0.53
	Dunkwa-on-Offin	39 ± 9	85 ± 5	0.23 ± 0.08	0.30 ± 0.09	0.05 ± 0.02	0.18
Central, Western, and Eastern	River Pra (Sediment)	38 ± 7	83 ± 4	0.22 ± 0.09	0.28 ± 0.05	0.05 ± 0.02	0.18
Eastern	New Abirim	18 ± 3	39 ± 5	0.11 ± 0.01	0.14 ± 0.02	0.03 ± 0.01	0.11
	Farmlands in New Juaben Municipality	21 ± 5	44 ± 10	0.12 ± 0.03	0.15 ± 0.03	0.03 ± 0.01	0.11
Greater Accra	Tema Metropolis	16 ± 3 29 ± 4^a^	35 ± 6 65 ± 8^a^	0.10 ± 0.02 0.18 ± 0.02^**a**^	0.11 ± 0.02 0.21 ± 0.02^a^	0.02 ± 0.01 0.04 ± 0.01^a^	0.07 0.14^a^
	Beach sand along the coastal belt of Greater Accra Region	40 ± 4	89 ± 8	0.24 ± 0.02	0.30 ± 0.03	0.05 ± 0.01	0.18
Western	Tarkwa	66 ± 11 617 ± 249^b^ 35±1^c^	137 ± 22 1392 ± 566^b^ 80±2^c^	0.37 ± 0.06 3.76 ± 1.52^b^ 0.22 ± 0.01^c^	0.48 ± 0.07 5.73 ± 2.17^b^ 0.32 ± 0.01^c^	0.08 ± 0.01 0.76 ± 0.30^b^ 0.04 ± 0.01^c^	0.28 2.66^b^ 0.14^c^
	Bibiani	20 ± 12	41 ± 17	0.11 ± 0.07	0.13 ± 0.08	0.03 ± 0.01	0.11
	Nkrofo	47 ± 7	101 ± 13	0.27 ± 0.03	0.30 ± 0.04	0.06 ± 0.01	0.21
	Bogosso-Prestea	78 ± 9	170 ± 18	0.45 ± 0.05	0.70 ± 0.07	0.10 ± 0.01	0.35
Upper East (sediment)	Tono Irrigation dam	23 ± 5	46 ± 10	0.13 ± 0.03	0.15 ± 0.03	0.03 ± 0.01	0.11
Average ± σ		**99 ± 31**	**149 ± 38**	**0.40 ± 0.11**	**0.56 ± 0.15**	**0.09 ± 0.03**	**0.30**
Range		**11–742**	**22–1392**	**0.06–3.76**	**0.07–5.73**	**0.02–0.76**	**0.07–2.66**
Limits [1,3]		**<57**	**<370**	**<1**	**<1**	**<0.07**^d^ **<1.5**^e^	**0.29**

*σ—Standard deviation*.

a *Soil from oil refinery*.

b *Oil & gas Sludges*.

c *Oil & gas Scales*.

d *Limit for Public exposure*.

e *Limit suitable for construction*.

Theoretically, the activity of U-238 is in equilibrium with Ra-226 in soil samples; hence, the estimated activity of U-238 can be utilized to assess the radiological hazard indices. The overall average value of the radium equivalent activity of the soil was found to be 149 ± 38 Bq/kg, ranging from 22 to 1392 Bq/kg. The corresponding absorbed dose rate, external hazard index, and internal hazard index were 99 ± 31 nGy/h (range: 11–742), 0.40 ± 0.11 (range: 0.06–3.76), and 0.56 ± 0.15 (range: 0.07–5.73), respectively.

The annual effective doses calculated are considered insignificant relative to the annual construction limit of 1 mSv/y, as detailed in Table 6. In recent years, the significance of using NORM residues, such as soils, in construction has grown, offering a way to reduce environmental NORM pollutants and promote sustainability. Given that NORM residues are often generated in substantial quantities, their use as mixtures or additive materials in building production could yield significant economic benefits while minimizing potential radiological impacts [61]. Literature suggests that mixtures or additives of NORM residues usually result in reduced activity concentration in the raw residue [[62], [63], [64], [65], [66], [67], [68], [69]]. There are potential opportunities to reuse these soils from the study areas as building materials. The majority of soil samples from this study are radiologically suitable for use as building materials, with their respective radiological hazard indices below the limits, except for the sludge and scale samples and soil samples from Ayanfuri and Bogosso-Prestea.

Therefore, it is recommended that sludges and scales be managed and regulated properly to avoid their inadvertent inclusion in building materials. These wastes should be safely stored or disposed of to allow for the terminal decay of all necessary radionuclide contents.

Table 7 displays the average activity concentrations of Ra-226, Th-228, and K-40 in the water samples, along with their annual committed effective doses. The overall average values for Ra-226, Th-228, and K-40 were 1.62 ± 0.33 Bq/L (range: 0.15–8.32 Bq/L), 2.08 ± 0.53 Bq/L (range: 0.19–8.70 Bq/L), and 22.36 ± 3.44 Bq/L (range: 0.34–166.74 Bq/L), respectively.

**Table 7:** Activity concentration of Ra-226, Th-228 and K-40 in water samples.

Region	Study area	Average and Range of Activity Concentration, Bq/L			Committed Effective Dose (mSv/y)	Excess Lifetime Cancer Risk (ELCR) 10^–3^
		Ra-226	Th-228	K-40		
Ashanti	Obuasi	0.32 ± 0.02 (0.15–0.46)	0.94 ± 0.05 (0.76–1.33)	0.69 ± 0.04 (0.52–0.87)	0.12 ± 0.01 (0.07–0.17)	0.41
	Konongo-Odumase	1.59 ± 0.51 (0.74–3.08)	4.44 ± 2.11 (2.71–11.79)	14.39 ± 3.76 (7.74–18.65)	0.62 ± 0.23 (0.33–1.33)	2.18
	Lake Bosomtwi, Kumasi	0.78 ± 0.02 (0.09–2.15)	0.19 ± 0.10 (0.03–0.86)	8.76 ± 2.40 (1.24–15.49)	0.21 ± 0.02 (0.03–0.55)	0.73
Brong Ahafo	Kenyasi	0.54 ± 0.03 (0.11–1.03)	0.41 ± 0.10 (0.21–0.56)	7.76 ± 2.70 (1.65–11.99)	0.17 ± 0.02 (0.04–0.29)	0.58
	Bui Dam	0.26 ± 0.01 (0.13–0.42)	0.47 ± 0.20 (0.07–0.82)	1.60 ± 0.30 (0.78–2.42)	0.09 ± 0.01 (0.07–0.14)	0.30
Central	Ayanfuri	0.16 ± 0.01 (0.11–0.23)	0.45 ± 0.08 (0.23–0.58)	0.75 ± 0.02 (0.64–0.89)	0.06 ± 0.01 (0.04–0.08)	0.21
	Dunkwa-on-Offin	4.72 ± 1.51 (1.01–7.03)	2.70 ± 0.40 (2.10–3.50)	53.90 ± 11.60 (19.80–62.20)	1.35 ± 0.38 (0.41–1.90)	4.73
Central, Western, and Eastern	River Pra	2.51 ±0.70 (1.38–4.01)	1.71 ± 0.49 (1.02–2.85)	41.43 ± 5.83 (29.40–47.77)	0.79 ± 0.20 (0.47–1.19)	2.77
Eastern	New Abirim	0.38 ± 0.06 (0.16–1.47)	0.55 ± 0.01 (0.12–2.21)	2.06 ± 0.43 (0.16–7.31)	0.12 ± 0.01 (0.04–0.45)	0.41
	Farmlands in New Juaben Municipality	0.23 ± 0.02 (0.18–0.29)	0.57 ± 0.04 (0.37–0.68)	0.34 ± 0.08 (0.25–0.48)	0.08 ± 0.01 (0.06–0.10)	0.27
Greater Accra	Tema Metropolis	0.15 ± 0.04 (0.05–0.22) 8.32 ± 1.56^a^ (7.08–12.42)	0.51 ± 0.04 (0.37–0.98) 8.70 ± 1.60^a^ (7.93–9.40)	0.35 ± 0.04 (0.21–0.52) 28.56 ± 7.20^a^ (26.94–31.70)	0.06 ± 0.01 (0.03–0.10) 2.29 ± 0.44^a^ (1.99–3.18)	0.21 8.00^a^
Western	Tarkwa	0.24 ± 0.04 (0.07–0.42)	0.90 ± 0.06 (0.65–1.12)	0.54 ± 0.05 (0.41–0.81)	0.10 ± 0.01 (0.05–0.15)	0.35
	Bibiani	0.86 ± 0.07 (0.13–2.87)	0.97 ± 0.33 (<MDA-4.29)	9.05 ± 1.45 (0.10–31.73)	0.27 ± 0.04 (0.03–0.96)	0.94
	Nkrofo	0.15 ± 0.03 (0.10–0.20)	0.98 ± 0.04 (0.70–1.52)	0.76 ± 0.06 (0.61–0.93)	0.09 ± 0.01 (0.06–0.12)	0.30
	Bogosso-Prestea	3.21 ± 0.44 (2.09–7.86)	6.40 ± 2.40 (3.12–9.58)	42.50 ± 8.00 (27.28–49.83)	1.18 ± 0.25 (0.71–2.34)	4.15
Upper East	Tono Irrigation dam	3.17 ± 0.57 (2.36–4.16)	4.46 ± 0.89 (2.68–5.48)	166.74 ± 14.55 (133.24–175.87)	1.66 ± 0.23 (1.23–1.93)	5.73
Average ± σ		**1.62 ± 0.33**	**2.08 ± 0.53**	**22.36 ± 3.44**	**0.54 ± 0.11**	**1.90**
Range		**0.15–8.32**	**0.19–8.70**	**0.34–166.74**	**0.06–2.29**	**0.21–8.00**
Guideline levels [1,3,29]		**10.0**	**1.0**	**N/A**	**0.1**	**1.16**

*σ*—*Standard deviation*.

a *Water from oil refinery site*.

It was observed that the concentration of U-238 in the water samples measured below the World Health Organization (WHO) recommended value. However, for Th-228, the overall average value exceeded the recommended concentration of 1.0 Bq/L. This discrepancy could be attributed to the relatively low solubility of thorium (Th) compared to uranium (U).

The higher concentrations of Th-228 compared to Ra-226 observed in this study might be attributed to the difference in solubility between thorium and uranium. Additionally, the fact that the water samples were not filtered before analysis could have influenced these findings. Thorium is known to be transported with particulate matter and tends to deposit in water bodies, thus the presence of particulate matter in the water samples could account for the elevated activity concentrations of Th-228. Understanding these factors is crucial for interpreting the observed radionuclide levels and their potential impacts on the environment and human health.

On the Excess Lifetime Cancer Risk estimation as observed from Table 6, Table 7, while specific sample location recorded extremely high Excess Lifetime Cancer Risk values some other sample location values were relatively low. The estimated ELCR values from the soil samples range from 0.07 × 10^−3^ to 2.66 × 10^−3^, with an average of 0.30 × 10^−3^, while the values estimated from the water samples ranges from 0.21 × 10^−3^ to 8.00 × 10^−3^, with an average of 1.90 × 10^−3^. The average ELCR values of the water samples is approximately about 1.6 times greater than the recommended values of 1.16 × 10^−3^ [1,3], and in some locations these values could even be 9 and 7 times higher than the recommended values both in soil and water respectively. The public in such sample’s locations with elevated ELCR values, have higher risk of developing cancer in these areas and there should not use the soils as building materials or drink from the water source.

Using data from Table 7, the descriptive statistics and the ANOVA tests for the three measured radionuclides in water samples across the regions shows in Table 8, Table 9, respectively.

**Table 8:** Activity concentration of Ra-226, Th-228 and K-40 in water samples.

Statistic Measurement	Ra-226 (Bq/kg)	Th-228 (Bq/kg)	K-40 (Bq/kg)	Committed Effective Dose (mSv/y)	Excess Lifetime Cancer Risk (ELCR)
Count	17	17	17	17	17
Mean	1.62	2.08	22.36	0.54	1.90
Std Dev	2.21	2.48	41.06	0.68	2.37
Min	0.15	0.19	0.34	0.06	0.21
25%	0.24	0.51	0.75	0.09	0.30
50% (Median)	0.54	0.94	7.76	0.17	0.58
75%	2.51	2.70	28.56	0.79	2.77
Max	8.32	8.70	166.74	2.29	8.00
Range	8.17	8.51	166.40	2.23	7.79
Variance	4.88	6.13	1686.16	0.46	5.63
IQR	2.27	2.19	27.81	0.70	2.47

**Table 9:** The ANOVA tests for each radionuclide in water samples across different region.

	Ra-226	Th-228	K-40
F-Value	0.089	0.128	4.453
P-Value	0.996	0.986	0.358

The high value of the average annual committed effective dose recorded in this study is likely due to most of the studies being conducted within mining communities, where illegal mining and pollution of water sources are prominent. As expected, water sampled from the oil refinery area showed high uranium and thorium contents. However, results were also high for the majority of the sample locations.

The average annual committed effective dose from the water concentrations was determined to be 0.54 ± 0.11 mSv/y, ranging from 0.06 to 2.29 mSv/y. Notably, the average annual committed effective dose (0.54 mSv/y) from the water samples is about five times higher than the WHO recommended guideline level of 0.1 mSv/y [70]. This indicates that the water sources in the study areas contain elevated levels of NORMs, posing significant radiation hazards to individuals consuming water from these sources if untreated.

In summary, the ANOVA tests indicate that for all the measured variables, there are no significant differences between the mean values of these variables across the different study areas. This uniformity implies that the study areas are relatively homogeneous in terms of these radioactive measurements and their potential health impacts. Also, it could be indicated that there is no statistically significant differences in the mean concentrations and committed doses among the study areas, as indicated by the high p-values.

Based on the nature of the data, multiple linear regression models were applied and The regression analysis results for each model are as follows:

*Predicting Ra-226 concentration based on Th-228 and K-40 concentrations:*

R-squared: 0.765 (This indicates that 76.5% of the variability in Ra-226 concentration can be explained by Th-228 and K-40 concentrations.). The coefficients suggest that both Th-228 and K-40 concentrations are positively correlated with Ra-226 concentration. Specifically, for every unit increase in Th-228 concentration, Ra-226 concentration increases by 0.745 units, and for every unit increase in K-40 concentration, Ra-226 concentration increases by 0.004 units.

*Predicting Th-228 concentration based on Ra-226 and K-40 concentrations:*

R-squared: 0.765 (This indicates that 76.5% of the variability in Th-228 concentration can be explained by Ra-226 and K-40 concentrations.). The coefficients indicate that both Ra-226 and K-40 concentrations are positively correlated with Th-228 concentration. Specifically, for every unit increase in Ra-226 concentration, Th-228 concentration increases by 0.935 units, and for every unit increase in K-40 concentration, Th-228 concentration increases by 0.005 units.

*Predicting K-40 concentration based on Ra-226 and Th-228 concentrations:*

R-squared: 0.240 (This indicates that 24.0% of the variability in K-40 concentration can be explained by Ra-226 and Th-228 concentrations.). The coefficients show a positive correlation between K-40 concentration and both Ra-226 and Th-228 concentrations. Specifically, for every unit increase in Ra-226 concentration, K-40 concentration increases by 4.660 units, and for every unit increase in Th-228 concentration, K-40 concentration increases by 4.247 units.

In conclusion, these results suggest that there are significant relationships between the concentrations of these radioactive elements in the soil samples. The high R-squared values in the first two models indicate a significant proportion of the variability in each element's concentration can be explained by the concentrations of the other two elements. The third model, however, has a relatively lower R-squared value, indicating a weaker predictive power. This analysis provides insights into the interrelationships of these elements, although it's important to note that causative mechanisms might be complex and could require more in-depth investigation beyond simple linear regression.

**Fig. 4:**
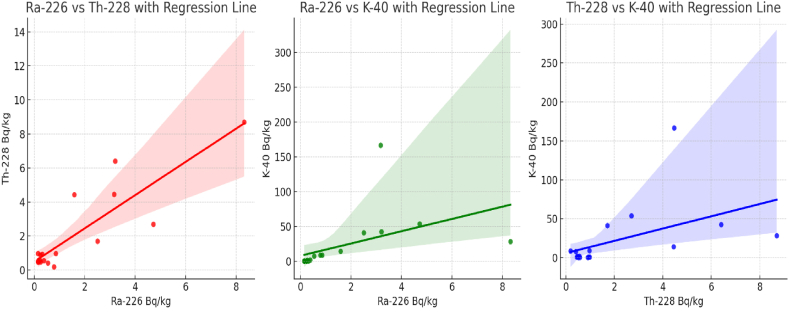
Scatter plot of radionuclides concentrations with the regression lines in water samples.

The correlations between pairs of radionuclides shows in Fig. 4, as these plots provide a visual representation of the relationships between different pairs of radionuclides, highlighting how their concentrations vary in relation to each other across different study areas.

**Fig. 5:**
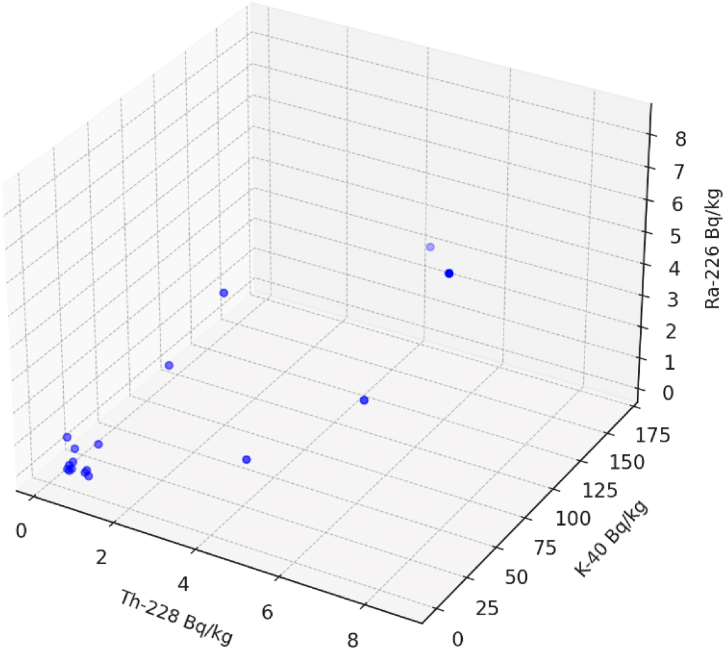
Scatter plot of radionuclides concentrations with the regression lines in soil samples.

The relationship between the concentrations of Th-228, K-40, and Ra-226 shows in Fig. 5. Each blue dot represents a data point from the dataset, with its position determined by the concentrations of Th-228 and K-40 on the x and y-axes, respectively, and the concentration of Ra-226 on the z-axis. Fig. 5 helps in understanding how the concentrations of Th-228 and K-40 together relate to the concentration of Ra-226 in your dataset.

While the majority of the water sources in the study areas are located in restricted areas and are not generally accessible for domestic purposes, it is imperative that surface water sources either remain under restrictive access or are properly treated for public use to mitigate potential health risks, similar to other study which was investigating the underground water quality from radiological point of view, in the same region, where influenced by mining activities and it could be found that The corresponding committed effective dose from consuming such groundwater was estimated to be higher than the recommended value [71].

It is observed from the tables that most of the studies were conducted in the Western Region of Ghana. This is attributed to the high concentration of mining and oil and gas companies in that part of the country. The other regions with significant studies include Ashanti, Eastern, and Central, as these areas also have abundant mineral resources. However, limited work has been done in the Greater Accra, Brong Ahafo, and Upper East regions, and no data is currently available for the other eight regions in Ghana. While these regions may not have extensive mining resources, it is important to establish base levels of NORMs to help estimate better regional and national levels for future radiological investigations and any required mitigation actions.

## 4 Conclusions

This study reports results on radioactivity concentrations of U-238, Th-232, and K-40 in soil and water in some mining communities in Ghana. The work considered public exposure to radiation from naturally occurring radioactive materials in soil and drinking water. The overall average concentrations of U-238, Th-232, and K-40 in the soil samples were calculated to be 59 ± 16 Bq/kg, 48 ± 15 Bq/kg, and 286 ± 57 Bq/kg, respectively. For the water samples, the overall average activity concentrations of Ra-226, Th-228, and K-40 were 1.62 ± 0.3 Bq/L, 2.08 ± 0.5 Bq/L, and 22.36 ± 3.4 Bq/L respectively. The average annual effective doses were estimated around 0.09 ± 0.03 and 0.54 ± 0.11 mSv/y for soil and water, respectively. The statistical analysis, encompassing descriptive statistics, ANOVA tests, and regression analysis, indicates no significant regional differences in the levels of radioactive elements. The regression models demonstrate strong interrelationships between the studied elements, with high R-squared values suggesting a predictable nature of one element’s concentration based on others. Nonetheless, the underlying causes of these correlations merit further exploration. The corresponding total annual effective dose was calculated as 0.63 ± 0.14 mSv per year. The total annual effective dose is below the 1 mSv per year dose limit recommended by the ICRP for public radiation exposure control. The average ELCR value of the water samples was 1.6 times greater than the recommended value of 1.16 × 10^−3^ which presents a relatively higher risk of the public developing cancer overtime. The high value recorded is expected since this report is data compiled on the status of NORMs studies carried out so far in the country with most of the studies carried out in gold mining and oil and gas companies that contain high uranium and thorium contents. In view of the high value recorded and that the total annual effective dose is approximately 1 mSv per year, it is recommended that the mining and oil companies establish a periodic (every 3 years) monitoring program for environmental radioactivity as the operations may alter the geochemical and radiological state of these mining communities. In conclusion, this study has provided a general picture of the status of NORMs studies conducted so far by establishing some regional data on NORMs in Ghana. The study showed that the activity concentrations and calculated doses from the investigated areas indicated significant levels of radiation exposure to the workers as well as members of the public. However, further studies need to be conducted in the Greater Accra, Brong Ahafo, Upper East, and the untouched eight other regions of Ghana to help establish a base level of NORMs. In order to better estimate a national level for future radiological investigation and mitigation actions that may be required.

## Data availability statement

All data presented at the article.

## CRediT authorship contribution statement

**Augustine Faanu:** Writing – review & editing, Writing – original draft, Validation, Supervision, Resources, Methodology, Funding acquisition, Conceptualization. **Lordford Tettey-Larbi:** Writing – review & editing, Writing – original draft, Methodology, Investigation, Formal analysis, Data curation, Conceptualization. **Esther Osei Akuo-ko:** Writing – review & editing, Methodology, Investigation. **Prince Kwabena Gyekye:** Writing – review & editing, Investigation. **David Okoh Kpeglo:** Writing – original draft, Validation, Supervision, Methodology, Formal analysis, Data curation, Conceptualization. **Henry Lawluvi:** Writing – original draft, Validation, Supervision, Methodology, Investigation, Data curation, Conceptualization. **Charles Kansaana:** Writing – review & editing, Writing – original draft, Methodology, Investigation, Formal analysis, Data curation. **Serwaa Adjei-Kyereme:** Writing – original draft, Methodology, Investigation. **Alexander Opoku Efa:** Writing – original draft, Methodology, Investigation. **Edit Tóth-Bodrogi:** Writing – review & editing, Data curation. **Tibor Kovács:** Writing – review & editing, Validation, Formal analysis, Data curation. **Amin Shahrokhi:** Writing – review & editing, Validation, Formal analysis, Data curation.

## Declaration of competing interest

The authors declare that they have no known competing financial interests or personal relationships that could have appeared to influence the work reported in this paper.
